# NiCrBSi Coatings Fabricated on 45 Steel Using Large Spot Laser Cladding

**DOI:** 10.3390/ma15186246

**Published:** 2022-09-08

**Authors:** Longjie Zhao, Huijun Yu, Yanxiang Wang, Zhihuan Zhao, Weihai Song, Chuanzhong Chen

**Affiliations:** 1 Key Laboratory of High Efficiency and Clean Mechanical Manufacture, Ministry of Education, and National Demonstration Center for Experimental Mechanical Engineering Education, School of Mechanical Engineering, Shandong University, Jinan 250061, China; 2 Key Laboratory for Liquid-Solid Structural Evolution and Processing of Materials, Ministry of Education, and Shandong Engineering & Technology Research Center for Superhard Material, School of Materials Science and Engineering, Shandong University, Jinan 250061, China; 3 School of Mechanical and Electronic Engineering, Shandong Agricultural and Engineering University, Jinan 250100, China

**Keywords:** nickel alloy, laser cladding, dendrites

## Abstract

Ni35 coatings were fabricated on 45 steel using a CO_2_ laser at various parameters. A relatively large spot (10 mm diameter) was adopted, which was beneficial to the coating quality and the cladding efficiency. The cross-sectional geometry, phase constituent, and microstructure of the coatings were investigated. With a lower specific energy, coating height increased, while coating width, melted depth, dilution rate, width to height ratio and contact angle decreased. Ni35 coating primarily consisted of γ-Ni, FeNi_3_, Ni_3_B, Cr_23_C_6_, and Cr_5_B_3_. Dendrites with flower-like, fishbone-like, pearl-like, and column-like morphologies were observed. The fraction of flower-like dendrites increased gradually with the decrease in scanning velocity due to the dendrite growth direction evolution. With the decrease in scanning velocity, the microstructure of the heat-affected zone transformed from martensite to martensite + sorbite and finally sorbite. The maximum microhardness of the Ni35 coating reached 451.8 HV_0.2_, which was about double that of the substrate (220 HV_0.2_).

## 1. Introduction

Generally, 45 steel is utilized to manufacture connecting rods, gears, and shafts owing to its high strength, good toughness, desirable welding performance, and low cost [1]. However, 45 steel components can be damaged under harsh service conditions, which increases the costs and reduces the production efficiency, or even leads to severe accidents. 45 steel hydraulic columns and cylinder rods require moderate hardness and good corrosion resistance.

In order to improve component performance, several surface modification technologies have been applied to 45 steel, including austenitic nitriding [2], physical vapor deposition (PVD) [3], plasma spray [4], and laser cladding [5,6]. Among these technologies, laser cladding has garnered increasing attention due to its unique advantages, such as high energy density, low dilution rate, narrow heat-affected zone, and metallurgical bonding to the substrate [7,8,9,10]. So far, researchers have made various attempts to enhance the surface property by laser cladding. Laser cladding coatings of NiCrBSi [11], NiMoCrSi [12], NiTiCr [13], and NiCrBSiC + WC [14] have been prepared on steel substrate.

NiCrBSi power is a popular cladding material due to its good self-fluxing capability, high wettability, and moderate price [15]. Owing to the intrinsic good toughness and reinforcement by borides and carbides, NiCrBSi coatings exhibit excellent mechanical properties. M.Q. Wan et al. [16] conducted a comparative study between Ni- and Fe- based laser cladding coating and found that Ni-based coating is a better choice in enhancing wear resistance as well as corrosion resistance. O.G. Devojno et al. [17] researched the influence of laser cladding conditions on the formation features, microstructure, phases, and microhardness of NiCrBSi coatings. Lino Costa et al. [18] proposed a semi-empirical method for laser cladding parameter selection through analysis of coating geometry under different process conditions.

In this research, Ni35 coatings were fabricated on 45 steel using a laser cladding process. Coating geometry was an important indicator of cladding quality. The effect of scanning velocity on the coating geometry was mainly discussed, which provided a reference for optimizing the processing parameters. The laser cladding process was modified in some aspects. Firstly, in order to control the preplaced thickness, a thin groove was machined on the substrate to contain the cladding powder. The preplacing method had advantages in the following aspects: (1) lower demand for powder flowability and (2) higher powder utilization compared to the synchronistic feeding method. Secondly, a relatively large laser spot (10 mm diameter) was applied instead of the small spot (3–5 mm diameter) generally used in past research [19,20,21,22,23]. At the same energy density, the energy input is increased with the increase in spot size, which decreases the cooling rate and reduces pores and cracks in the coatings. Additoinally, a large spot size is preferable in practical applications for the purposes of high efficiency. The phase constituent, microstructure, and microhardness of the coating were studied. Moreover, the morphologies of the heat-affected zone at various scanning velocities were analyzed, which has been rare in past research. The relevant discussion is helpful for understanding the microhardness changes in the depth direction.

## 2. Materials and Methods

### 2.1. Materials

45 steel with ferrite and pearlite microstructure was selected as the substrate. The steel was produced via a hot forging process and designated according to steel standard GB 699-88. Ni35 powder was employed as the cladding material. The powder was produced through gas atomization process and supplied by BGRIMM Advanced Materials Science and Technology Co., Ltd. (Beijing, China). The powder particles were spherical or nearly spherical with the particle diameter of 45–109 μm. The chemical compositions of Ni35 powder and 45 steel are shown in Table 1 and Table 2, respectively.

Using wire cutting, 45 steel plates were cut to a size of 100 × 22 × 12 mm^3^ and machined with a milling machine to remove the oxide skin. A groove of 1.1 mm depth was machined subsequently with a milling machine on the plane of 100 × 22 mm^2^. Next, the substrate was cleaned with alcohol using an ultrasonic cleaner and air dried.

### 2.2. Powder Preplacing and Laser Cladding

**Figure 1:**
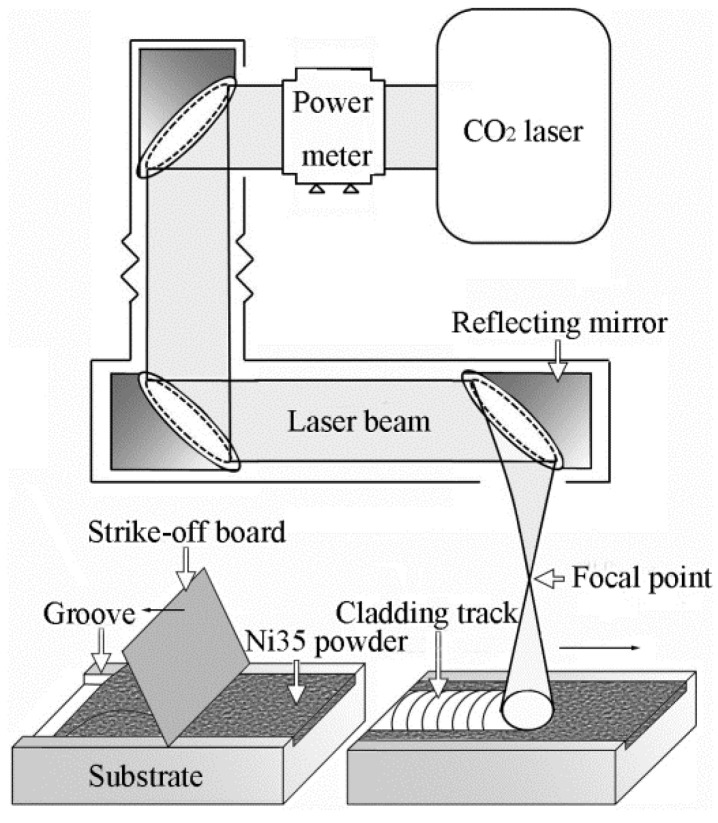
Schematic illustration of the preplacing method and laser cladding process.

The preplacing method is shown in Figure 1. Ni35 powder was placed into the groove and then smoothed using a strike-off board. By this means, the thickness of the preplaced powder was controlled at 1.1 mm by the groove. No binder was utilized to avoid pores or composition pollution caused by binder decomposition.

A TEL-6K CO_2_ laser with the intensity distribution of the Gaussian type was applied. Prior to the laser cladding process, the laser spot size was defined by measuring the burn track on 45 steel plates. The laser head was raised gradually to increase the defocusing distance and obtain a larger spot size until a diameter of 10 mm was obtained. Subsequently, the preplaced Ni35 powder on the substrate was scanned by the laser beam, as shown in Figure 1. The laser powers (P) employed in this experiment were 4, 4.5, and 5 kW. Scanning velocities (Vs) were 100, 200, and 300 mm/min. No shielding gas was applied for fear of blowing the powder away.

### 2.3. Characterization of the Coatings

After the laser cladding process, the specimens were cut at the half-length position via wire cutting. Then, the cross section of specimens was polished and etched using a solution of 20 vol.% HF + 30 vol.% HNO_3_ + 50 vol.% HCl. The etched specimens were examined using an optical microscope (OM), and the cross-sectional geometry parameters, including cladding height (H), melt depth (D), and coating width (W), were measured using ImageJ 1.40 software.

The coating surface was ground with sandpaper to remove the oxidized layer and then scanned using the D/max-RC X-ray diffractometer to analyze the phase constituents. The coating microstructure was observed using a JSM-6380LA scanning electron microscope (SEM) equipped with an energy dispersive spectrometer (EDS) (JEOL, Akishima City, Japan). The microstructure of the heat-affected zone (HAZ) was observed via OM.

The microhardness distribution on the cross section was tested with an HV-1000 microhardness tester. Indentations were performed with a 0.15 mm interval distance. The applied load for the microhardness test was 200 gf, and the holding period was 10 s.

## 3. Results and Discussion

### 3.1. Coating Geometry

**Figure 2:**
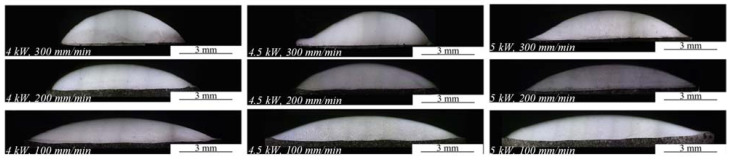
Cross-sectional morphology of Ni35 coatings.

**Figure 3:**
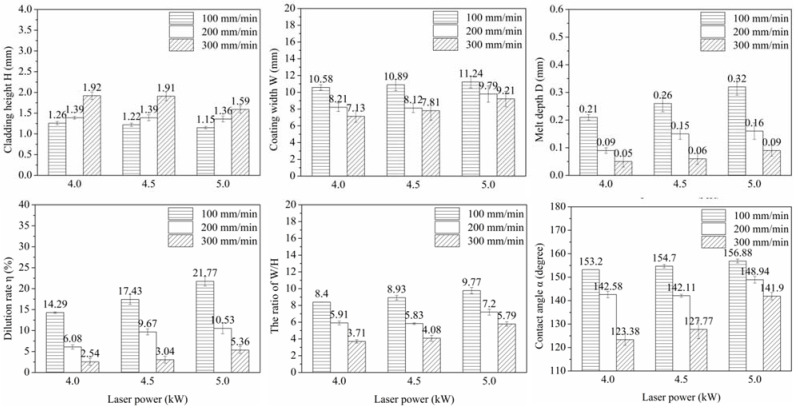
Geometry parameters of Ni35 coatings.

Figure 2 shows the cross-sectional morphology of Ni35 coatings. The geometry of the coatings was measured and plotted in Figure 3. It was noted that H ranged from 1.15 mm to 1.92 mm, which exceeded the preplaced powder thickness (1.1 mm). During laser cladding, the molten pool surface had a contraction tendency due to the surface tension effect. A liquid flow moved towards the molten pool top, which led to the arcuate coating surface and increased H. Additionally, Ni35 has good wettability with the substrate, which restrains powder spatter and decreases mass loss in laser cladding to increase H. H increased with a higher Vs or lower P. As is known, the specific energy (Es) in the laser cladding process is defined as Es = P/(Φ·Vs) [24,25], where Φ represents the laser spot diameter. With a higher Vs or lower P, the Es decreased. Consequently, the temperature and wettability of the melted powder were lowered due to the decreased energy input, which inhibited the liquid spread and raised H. The effect of scanning velocity on H is more remarkable than that of laser power. This is because that scanning velocity has a larger influence on Es than laser power in the processing parameter range. W decreased with a higher Vs or lower P due to the inhabited liquid spread at a lower Es. It was reasonable that W showed an opposite trend compared to H since the amount of the preplaced powder was fixed. However, there was an exception for the coatings fabricated at 4 and 4.5 kW, 200 mm/min. W decreased from 8.21 mm to 8.12 mm with P increasing from 4 and 4.5 kW. This might be due to the fluctuation of the cladding track width. D became lower with the increase in Vs or decrease in P. This was because less substrate was melted when the Es decreased.

In the laser cladding process, the composition of the coating is impacted by the slightly melted substrate. The extent of the impact can be represented by the dilution rate (η), which is calculated as η = D/(H + D) [26,27]. In general, η of 5–8% was regarded as acceptable to control the composition alteration and guarantee the coating property. In the present research, desirable η values were obtained at 4 kW, 200 mm/min and 5 kW, 300 mm/min, which were 6.08% and 5.36%, respectively. Additonally, the ratio of W/H and the contact angle α, in which α = 180 − 2 arctan (2 H/W), were calculated. A high W/H is conducive to increasing the process feasibility of multi-track laser cladding and eliminating the overlapping pores. A high contact angle α reflects the good wettability between the coating and substrate. Both W/H and the contact angle α increased with a lower Vs or higher P.

### 3.2. Coating Phase Constitution

**Figure 4:**
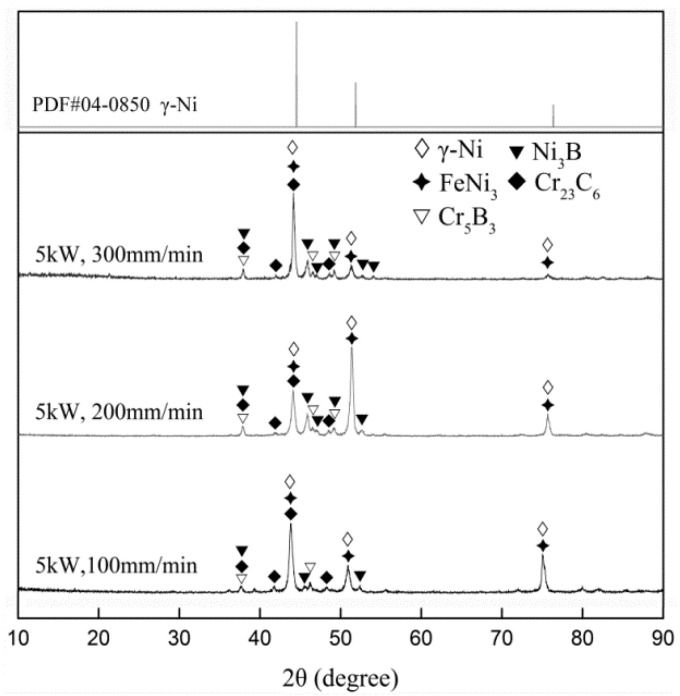
XRD patterns of γ-Ni and Ni35 coatings.

As shown in Figure 4, Ni35 coatings prepared at different Vs had analogous phase constitution, which was mainly composed of γ-Ni, FeNi_3_, Ni_3_B, Cr_23_C_6_, and Cr_5_B_3_. Compared to the standard γ-Ni peaks, γ-Ni peaks of the coatings shifted left slightly, indicating that the lattice parameter was enlarged with reference to the Bragg equation. This result corresponded to the observations in the research of T.E. Abioye [28]. This was due to the dissolution of Fe in γ-Ni because of the high element affinity between Fe and Ni [29]. With the decrease in Vs, due to the increased dilution rate η, more Fe atoms entered the molten pool and dissolved in γ-Ni. As a result, the γ-Ni peaks of the coatings moved further to the left.

### 3.3. Coating Microstructure

**Figure 5:**
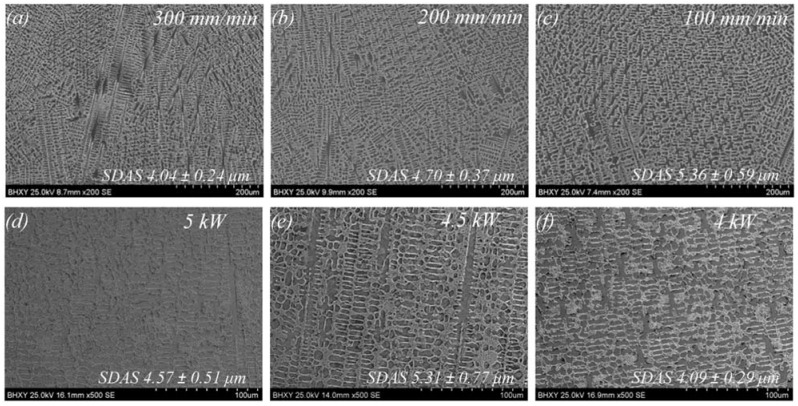
SEM morphologies of Ni35 coatings fabricated at different processing conditions: (**a**) 5 kW, 300 mm/min; (**b**) 5 kW, 200 mm/min; (**c**) 5 kW, 100 mm/min; (**d**) 5 kW, 200 mm/min; (**e**) 4.5 kW, 200 mm/min; and (**f**) 4 kW, 200 mm/min.

The microstructure of Ni35 coatings fabricated at different Vs and P is shown in Figure 5. Generally, the microstructure was composed of dendrites and interdendritic networks, which exhibited a typical rapid solidification feature. A similar microstructure composed of dendritic γ-Ni and Ni/Ni_3_B eutectic was observed in the research of Zoran Bergant et al. [30]. The volume fraction of dendrite was about 56% for coating fabricated at 5 kW, 200 mm/min. No pores or cracks were found, demonstrating a desirable coating quality. The secondary dendrite arms spaces (SDAS) are measured through SDAS = L/(N − 1), in which L is the test line length and N is the number of secondary dendrite arms [31]. According to Figure 5a–c, the average SDAS of Ni35 coatings were 5.36, 4.70 and 4.04 μm for P of 5 kW and Vs of 100, 200 and 300 mm/min, respectively. With the increase in Vs, the solidification rate of the molten pool was increased. As a result, the dendrite growth was inhabited due to the limited liquid period, causing a lower SDAS.

With the same Vs of 200 mm/min, the average SDAS were 4.09, 5.31, and 4.57 μm for P of 4, 4.5, and 5 kW, respectively, as is shown in Figure 5d–f. The results showed that laser powder had a nonmonotonic effect on the microstructure refinement, which was different from the scanning velocity. It is known that the extent of the microstructure refinement depends on the cooling rate of the molten pool. The fine microstructure is obtained with a rapid cooling rate. Under the condition of 4 kW, the majority of the laser energy was absorbed by the cladding powder. Only a small fraction of the energy was transported to the substrate. Thus, the temperature gradient between the molten pool and the substrate was relatively remarkable, leading to the rapid cooling rate and hence the fine SDAS. When the laser power increased to 4.5 kW, the laser energy conducted to the substrate increased, which decreased the temperature gradient and reduced the cooling rate, resulting in the coarse SDAS. Nevertheless, the SDAS decreased with the further increase in the laser power from 4.5 to 5 kW. This was due to the increased cladding width at 5 kW, as was discussed in Section 3.1, which accelerated the cooling process and decreased the SDAS.

**Figure 6:**
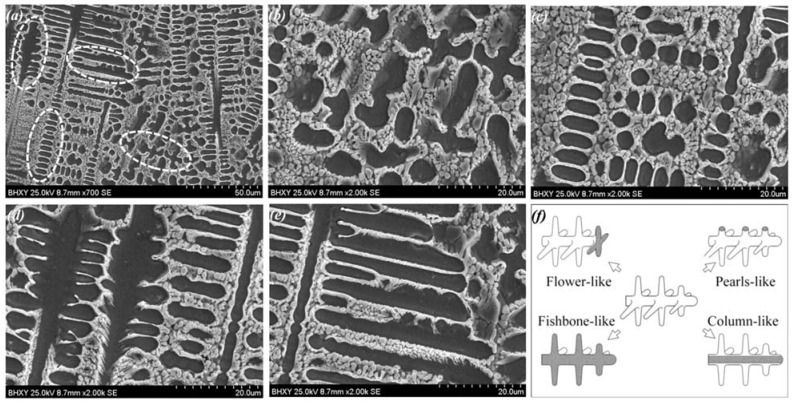
SEM morphologies of the Ni35 coatings fabricated at 5 kW, 200 mm/min: (**a**) various dendrite morphologies, (**b**) flower-like morphology, (**c**) pearl-like morphology, (**d**) fishbone-like morphology, (**e**) column-like morphology, and (**f**) schematic illustration of the sectioning effect.

Figure 6 shows the various morphologies of dendrites in Ni35 coating (5 kW, 200 mm/min). Flower-like, fishbone-like, pearl-like, and column-like morphologies were observed. This was because of the complex dendrite growth direction and the sectioning effect, which are illustrated briefly in Figure 6f. As is shown, flower-like morphology appeared when the growth direction was perpendicular to the cross-section. Pearl-like morphology represents an array of the secondary dendrite arms. Fishbone-like morphology is shown when the dendrite is sectioned along its symmetry plane.

As shown in Figure 5a–c, the fraction of flower-like dendrites increased gradually with the decrease in Vs, which reflected the growth direction evolution. With the high Vs of 300 mm/min, the melted depth D was low, as mentioned above. The bottom region of the molten pool solidified rapidly due to the low D and high Vs, which facilitated the heat dissipation from the molten pool to the beneath substrate [32]. As a result, dendrites with a vertical growth direction were dominant [33], which displayed fishbone-like or column-like morphologies on the cross-section. With the decrease in Vs, heat flow turned to the molten pool tail. As a result, the dendrite growth direction became perpendicular to the cross-section, and more flower-like dendrites appeared.

**Figure 7:**
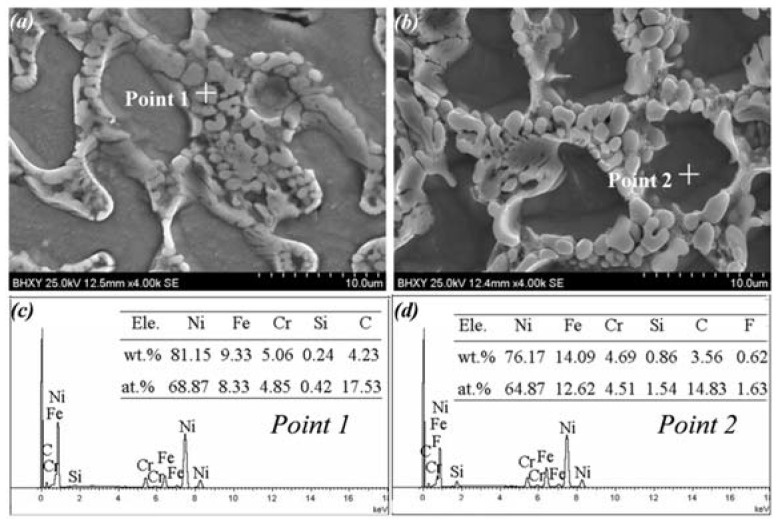
SEM morphologies and EDS analysis of the Ni35 coatings fabricated at 5 kW, 200 mm/min: (**a**,**b**) SEM morphology, (**c**) EDS result of point 1, and (**d**) EDS result of point 2. (EDS test position indicated by the cross in (**a**,**b**)).

According to the EDS results in Figure 7, the interdendritic zone (point 1) contained higher Ni, Cr, and C and lower Fe contents. With reference to the XRD analysis and previous research [34,35,36], it was inferred preliminarily that Ni_3_B, Cr_5_B_3_, and Cr_23_C_6_ existed in the interdendritic zone. The dendrite zone (point 2) contained high Ni and Fe contents, which might consist of γ-Ni and FeNi_3_.

### 3.4. The Microstructure Evolution of HAZ

**Figure 8:**
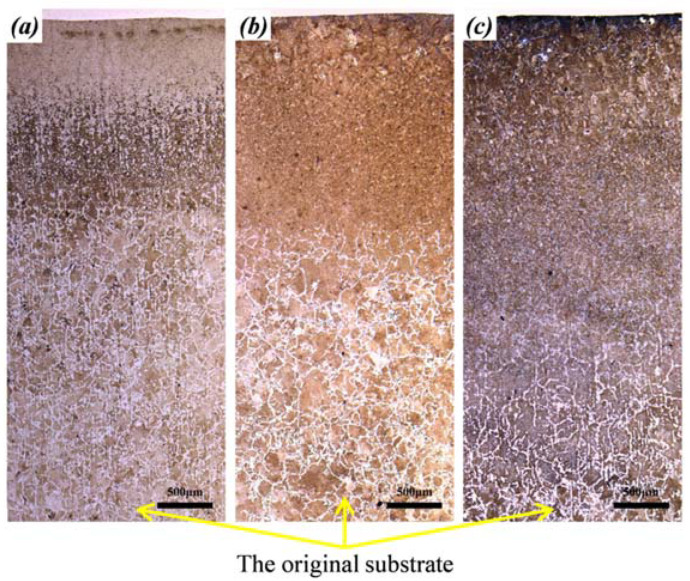
Macroscopic views of the HAZ at 5 kW: (**a**) 300 mm/min, (**b**) 200 mm/min, and (**c**) 100 mm/min.

Figure 8 shows the macroscopic views of HAZ at different Vs. It was noted that the HAZ depth increased with the decrease of the Vs from 300 mm/min to 100 mm/min. This was attributed to the fact that the substrate obtained more laser energy at lower Vs. As the laser power decreased from 5 kW to 4 kW, the HAZ depth decreased. This was because the substrate obtained less laser energy at lower laser power. The microstructure evolution from HAZ to the original substrate was exhibited, which resulted from the energy gradient along the depth direction. With the increase in depth, the heating temperature decreased gradually, causing different phase transformations in different locations. In general, HAZ was composed of a quenching zone, a normalizing zone, and an annealing zone from the top to the bottom. As was shown in Figure 8a, the typical characteristics were observed in HAZ formed at 5 kW, 300 mm/min, whereas the quenching zone was suppressed for HAZ at 200 and 100 mm/min, as shown in Figure 8b,c. This was because the lower Vs reduced the cooling rate and consequently inhibited the quenching process. In addition, as was shown in the bottom location of the macroscopic views, the steel substrate without phase transformation consisted of ferrite and pearlite.

**Figure 9:**
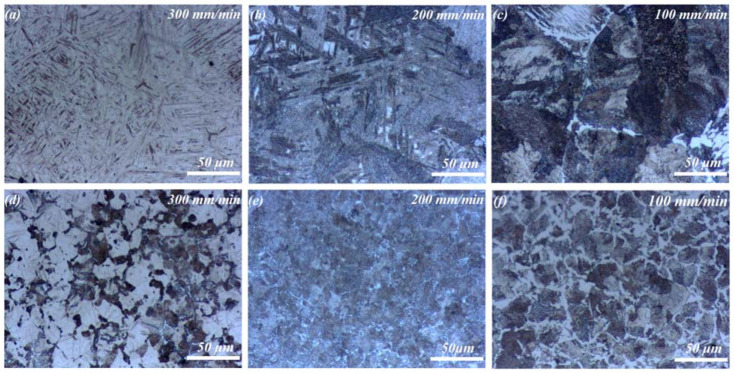
Optical morphologies of the HAZ at different scanning velocities, 5 kW: (**a**–**c**) upper region of the HAZ and (**d**–**f**) lower region of the HAZ.

Due to laser irradiation and heat conduction, the substrate temperature was raised rapidly above Ac_3_ (the temperature at which the phase constituent of the hypoeutectoid steel is completely transformed to austenite during the heating process), inducing the austenization of 45 steel. Subsequently, the substrate underwent rapid cooling and transformed to martensite, as shown in Figure 9a, whereas as the depth increased, the available heat declined, leading to the incomplete austenization. An abnormal quenching microstructure consisting of martensite and troostite was formed, as shown in Figure 9d.

As Vs decreased to 200 mm/min, the cooling rate of the substrate was decreased. In this condition, sorbite transformation occurred partially, and the mixed microstructure of martensite and sorbite formed, as shown in Figure 9b. Additionally, the martensite at 200 mm/min became coarser compared to that at 300 mm/min. As the depth increased, the microstructure consisted of sorbite, as shown in Figure 9e.

As Vs decreased to 100 mm/min, the cooling rate decreased further. As a result, the martensite transformation was restrained, and the normalizing microstructure of sorbite was observed, as shown in Figure 9c. Moreover, due to the huge heat input, Widmanstätten ferrite was observed, and the austenite grain was coarse [37]. In the upper region, the austenite grain was relatively coarse. This was because that the formation temperature of austenite was relatively high in the upper region during laser cladding [38]. However, with the increase in depth, the austenite grain was refined due to the decreased formation temperature of austenite, as shown in Figure 9f.

### 3.5. Microhardness

**Figure 10:**
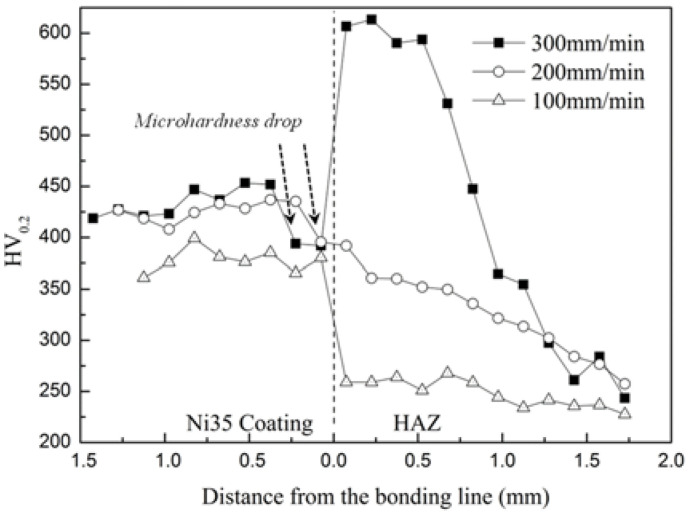
Microhardness distribution of Ni35 coatings fabricated at 5 kW.

Figure 10 depicts the microhardness distribution from the coating surface to HAZ. The average microhardnesses of the coatings were 426.5 ± 21.7 HV_0.2_, 423.0 ± 13.6 HV_0.2_, and 378.0 ± 11.7 HV_0.2_ for Vs of 300, 200, and 100 mm/min, respectively. It was demonstrated that the microhardness increased with the increase in Vs. This was ascribed to the accelerated solidification at higher Vs, which decreased the grain size and improved the microhardness. The maximum microhardness of Ni35 coating reached 451.8 HV_0.2_, which was approximately 2 times that of the substrate (220 HV_0.2_). This was because of the high hardness of Ni alloy and the reinforcement effect of Ni_3_B, Cr_23_C_6_, and Cr_5_B_3_. It was noted that at 5 kW and 300 mm/min, the microhardness of the HAZ reached approximately 600 HV_0.2_ near the bonding line. The significant enhancement of the microhardness is ascribed to the martensite transformation in the HAZ at 5 kW and 300 mm/min, as discussed in Section 3.4.

As indicated by the arrow, a microhardness drop occurred in the bottom region of the coating for Vs of 300 and 200 mm/min. This was because the martensite transformation could result in volume expansion in the HAZ, which caused tensile stress in the adjacent coating bottom. Moreover, microhardness decreased due to the existence of tensile stress. This was absent for Vs of 100 mm/min due to the absence of martensite transformation.

**Figure 11:**
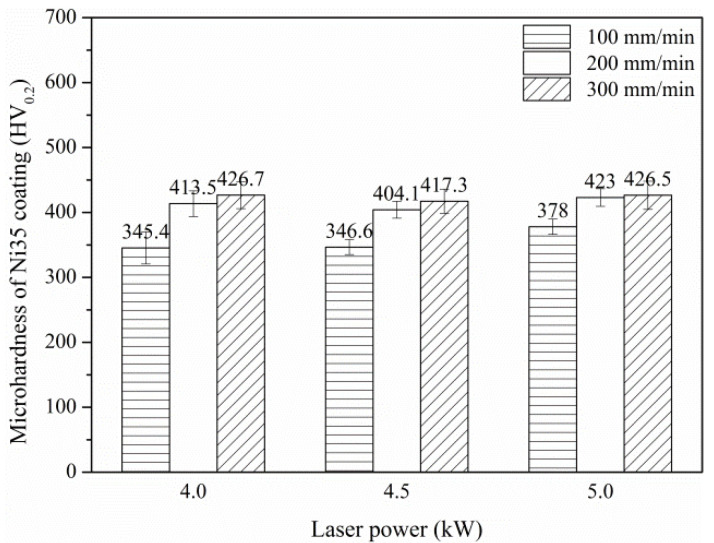
Average microhardness of Ni35 coatings.

The average microhardness values of the Ni35 coatings were shown in Figure 11. It was found that for a given laser power, microhardness increased gradually with the increase in Vs from 100 mm/min to 300 mm/min. This was ascribed to the finer microstructure at the higher Vs, which resulted from the lower laser energy input. However, the effect of laser power on the average coating microhardness was limited. This is because that the effect of laser power on the specific energy is slight for the given levels in the range of 4–5 kW.

As mentioned in Section 3.1, the processing conditions for the desirable η were 4 kW, 200 mm/min and 5 kW, 300 mm/min. As seen in Figure 11, the average coating microhardnesses were 413.5 and 426.5 HV0.2 for 4 kW, 200 mm/min and 5 kW, 300 mm/min, respectively. Hence, the optimal processing conditions were selected at 5 kW, 300 mm/min.

## 4. Conclusions

Ni35 coatings were fabricated on 45 steel via the large spot laser cladding process. With a higher scanning velocity or lower laser power, the cladding height H was increased, while the coating width W, melt depth D, dilution rate η, W/H ratio, and contact angle α were decreased. The geometry parameters of Ni35 coatings could be controlled by adjusting the specific energy Es. The phase constituent of Ni35 coatings primarily consisted of γ-Ni, FeNi_3_, Ni_3_B, and Cr_23_C_6_. The microstructure consisted of dendrites and interdendritic networks. Dendrites exhibited various morphologies due to the complex growth direction and the sectioning effect. The change of Vs altered the heat flow in the molten pool and led to the dendrite growth direction evolution. The microstructure of HAZ varied with scanning velocity and depth.

Compared to the substrate, Ni35 coatings had an enhanced microhardness because of the high hardness of Ni alloy along with the reinforcement effect of Ni_3_B, Cr_23_C_6_, and Cr_5_B_3_.

## Figures and Tables

**Table 1:** Chemical composition of Ni35 powder.

Element	C	B	Si	Cr	Fe	Ni
wt.%	≤0.25	1.5~3	2.5~4	9~12	≤14	Bal.

**Table 2:** Chemical composition of 45 steel.

Element	C	Cr	Mn	Ni	Si	P	S	Fe
wt.%	0.42~0.50	≤0.25	0.50~0.80	≤0.25	0.17~0.37	≤0.035	≤0.035	Bal.

## Data Availability

The data presented in this study are available on request from the corresponding author.
